# Comparison of Goslon Yardstick and Cephalometric Analysis in Nonsyndromic Unilateral Cleft lip and Palate Individuals in the Western Cape, South Africa

**DOI:** 10.1177/10556656251385296

**Published:** 2025-10-21

**Authors:** Albert M. van Zyl, Haydn H. Bellardie

**Affiliations:** 1 Department of Orthodontics, Faculty of Dentistry, 72027 University of the Western Cape, Mitchells Plain, South Africa

**Keywords:** cephalometrics, cleft lip and palate, craniofacial growth, craniofacial morphology, dental arch relationship, Goslon Yardstick, maxillomandibular relationship, orthodontics, sagittal jaw relationship, treatment outcome, unilateral cleft lip and palate, maxilla growth, midfacial growth

## Abstract

**Objective:**

To establish correlations between skeletal jaw relationship measured on lateral cephalograms and Goslon Yardstick scores for dental arch relationship (DAR) on orthodontic study models for unilateral cleft lip and palate (UCLP).

**Design:**

Retrospective review of consecutive cases.

**Setting:**

Multidisciplinary cleft and craniofacial clinics at two tertiary care centers in the Western Cape, South Africa.

**Patients:**

Forty-nine consecutive patients with nonsyndromic UCLP before they received orthodontic treatment and secondary alveolar bone graft (SABG).

**Interventions:**

Fourteen cephalometric angles measured by two observers and Goslon Yardstick scores determined by three observers. Inter- and intraobserver reliability determined using Cohen's Weighted Kappa statistic.

**Main Outcome Measures:**

Age, gender, and side of cleft were recorded. Cephalometric measurements and Goslon scores compared with regression analysis to determine correlations between angle ANB (cephalometric angle indicating anteroposterior relationship between the maxilla and mandible) and Goslon scores.

**Results:**

Mean age 10.7 years; 22 males and 27 females. Thirty-four (69.4%) of the clefts were left-sided. Kappa statistics ranged from good to very good for inter- and intraobserver reliability for cephalometric measurements and Goslon scores. No statistically significant differences between genders for cephalometric measurements and Goslon scores (*P* > .05). Mean ANB = 0.2(2.39) indicates Class III skeletal relationships for these individuals. Mean Goslon score 2.89. There was a moderate negative correlation of *r* = −0.5691 (*P* = 0) between ANB and Goslon score.

**Conclusion:**

Moderate negative correlation between ANB and Goslon Yardstick provides evidence that Goslon scores are valid and reliable indicators of skeletal jaw discrepancy for UCLP without the errors encountered using cephalometric radiographs.

## Introduction

Orofacial clefts (OFCs) are the most common congenital disorders of the head and neck, and affect approximately 1 in 700 live births globally.^1^ In South Africa (SA) the estimated prevalence of cleft lip and/or palate (CLP) in the public health sector is 0.1/1000 to 1.2/1000 live births, after adjusting the denominator for missing data and live births occurring in the private health sector.^2^

As with other cleft types, individuals with nonsyndromic unilateral cleft lip and palate (UCLP) require treatment by a multidisciplinary team of health professionals and involvement of families and caregivers in order to achieve the best quality of life for them.^3^ The multidisciplinary team should become involved in the care of the individuals and their families as early as possible, even before birth, to organize a comprehensive and well-planned approach to the complex medical, dental and surgical needs of these individuals into adulthood.^4^ Centralized cleft teams ideally comprise geneticists, surgeons, speech therapists, dietitians, psychologists, orthodontists and dentists.^5–8^

Based on research conducted at 11 specialist academic centers in six of the nine provinces in SA, Hlongwa and Rispel^9^ conceptualized the Ekhaya Lethu (isiZulu meaning “House of Care”) model for the management of CLP care in SA and other low- and middle-income countries.

The International Committee on Cleft Documentation and Measurements has recommended that records of CLP patients be taken at the age of five years,^10^ and some research has indicated that treatment outcome can be predicted in UCLP patients when they are 5 years old.^11^ Semb^12^ and Mars et al.^13^ have reported that midface growth is probably an acceptable indicator of surgical outcome.

Cephalometric analysis is used extensively to evaluate the craniofacial morphology in individuals with UCLP. Several intercenter studies have used changes in cephalometric parameters during growth and after various surgical procedures as an outcome measure (Scandcleft[SC],^14^ Slavcleft,^15^ Americleft^16^ and Eurocleft^17,18^). Angle ANB is a cephalometric angle indicating anteroposterior relationship between the maxilla and mandible; it is the angle measured between A (subspinal point), N (nasion) and B (suprementale) on cephalometric tracings.^14^ There is a need for standardization of specifications of cephalometric equipment to reduce errors such as variable magnification and distortion of sections of images.^17,18^ Using an expert digitizer/s is important in reducing the random error of the method.^16^

Various indices have been used to analyze the dental arch relationship (DAR) in individuals with UCLP in an attempt to measure treatment outcome more accurately.^19–21^ In a literature review of electronic databases from 1987 to 2013, Haque et al.^20^ concluded that the Goslon Yardstick, five-year-old yardstick, EUROCRAN Yardstick, Huddert–Bodenham index and modified Huddart-Bodenham (MHB) index can be used to assess DAR in individuals with UCLP. The Goslon (Great Ormond Street, London and Oslo, Norway) Yardstick is the most commonly used index.^20^ It was developed to categorize the degree of malocclusion in 10-year-old children with UCLP, during the late mixed dentition or early permanent dentition stages.^13,21,22^ The scoring system ranks the degree of malocclusion and prospects for orthodontic and surgical correction of the malocclusion. Conventional plaster study models or 3D digital orthodontic study models are considered to be equally reliable to use for DAR measurements.^23^

The three clinical features that the researchers considered to be crucial for categorizing malocclusions in the early permanent dentition of children with UCLP are the anteroposterior arch relationships, vertical labial segment relationships and transverse relationships.^24^

The comparative effectiveness of using the Goslon Yardstick to determine DAR and lateral cephalometric analysis to assess craniofacial form in assessing outcomes of individuals with UCLP remains largely unexplored. The main objective of this study was to determine the correlation between cephalometric parameters of sagittal jaw relationship and DAR measured by the Goslon Yardstick in a group of individuals with UCLP.

## Methods

### Study Design and Patient Selection

A retrospective descriptive and inferential study was conducted to assess craniofacial morphology using lateral cephalograms and DAR using the Goslon Yardstick, and to determine the correlation between these measurements for the individuals with UCLP. Ethics approval was granted for this research project.

The objectives of the study were to determine the craniofacial morphology of individuals with UCLP using cephalometric parameters of skeletal, dentoalveolar and soft tissue measurements, to determine the DAR of these individuals using the Goslon Yardstick and to determine the correlation between the cephalometric parameters of craniofacial morphology and the Goslon Yardstick scores for DAR for these individuals.

Inclusion criteria were individuals with UCLP who did not have craniofacial syndromes or Simonart's bands, who had not undergone orthodontic intervention or SABG, whose cephalograms and study models were taken at the same time, and whose cephalograms and study models were of good quality. The records of individuals with UCLP who had craniofacial syndromes or Simonart's bands, who had received any orthodontic treatment or SABG, whose records were not taken at the same time or were of substandard quality, were excluded from the study.

A consecutive sample of 49 seven- to 14-year-old individuals with UCLP who met the inclusion and exclusion criteria was retrospectively collected using the archived records from two multidisciplinary tertiary care centers. The centers are located within the same city and patients are shared between the two centers. Demographic information, lateral cephalometric radiographs and study models were retrieved and analyzed.

### Data Collection

Permission to take the records was received when patients opened files at the centers. No name or any other identifying information was available on the records when the research was done. A number was assigned to each study model and cephalogram. All identifiers were stored separately and were only accessible to the researcher. All data collected during the study was stored securely on a password-protected device.

### Cephalometric Measurements

Analogue and digital lateral cephalograms taken at the same time as the orthodontic study models were retrieved from the patient records.

The lateral cephalograms were converted into digital format and subsequently traced using the Dolphin software program (Dolphin Imaging and Management Solutions, Chatsworth, CA, USA) by two observers. A scaled calibration was performed before landmark identification was done. Image-enhancing techniques including brightness, contrast modification, and magnification were used to enhance landmark identification.

**Figure 1.**
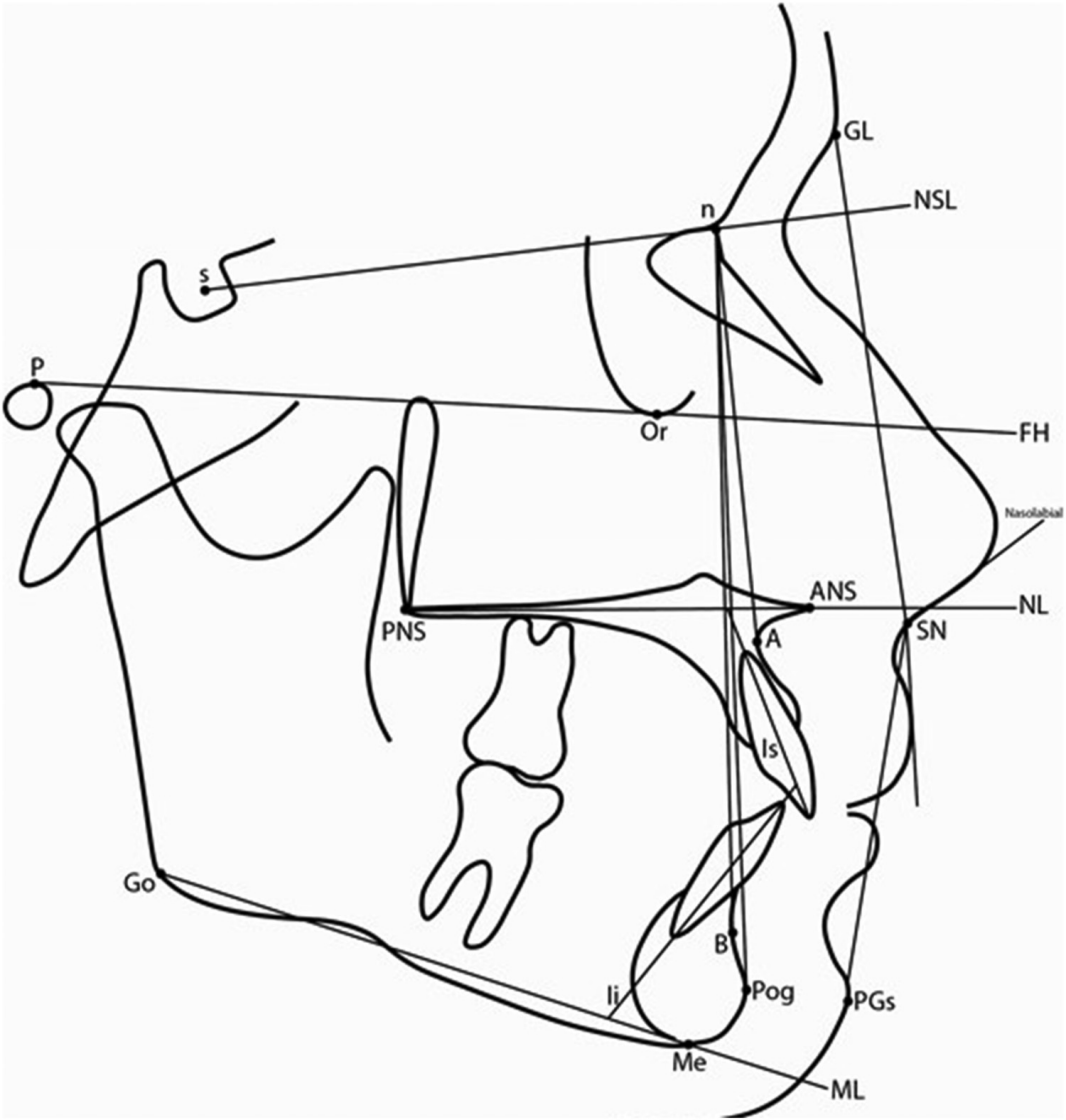
Reference Point and Lines on the Tracing of the Lateral Cephalogram and the Measured Angles.

Fourteen cephalometric parameters described in the SC study were used in this study (Figure 1).^14^

Copyright obtained for Figure 1 in Kuseler et al.;^14^ Oxford University Press, License Number 6058811342341. Skeletal reference points: *s,* sella; *n,* nasion; ANS, anterior nasal spine; *A,* subspinal point; *B,* supramentale; PNS, posterior nasal spine; Pog, pogonium; *P,* porion; Or, Orbitale; Me, menton; Go, gonium; GL, soft tissue glabella; SN, subnasale; PGs, soft tissue pogonion. Lines: NSL, nasion-sella-line; FH, Frankfurt horizontal line; NL, nasal line, ML, mandibular line; Is, axis of the upper incisor; Ii, axis of the lower incisor. Measured angle: SNA, SNB, SNPog, ANB, ANPog, NSL/NL, Is/FH, Is/ML, Is/Ii, Nasolabial angle = angle between a line from SN and tangent to the lower border of the nose and from SN and tangent to the upper lip, Soft tissue profile = GL-SN-PGs.

To exclude radiographic error due to the use of different cephalometric equipment, only angular measurements were analyzed, with no linear measurements included in the analyses. Using the cephalograms produced by various types of radiographic equipment would incorporate errors from differential magnification of the radiographs. The cephalometric tracings were done, 10 at one sitting, by each of the examiners on the Dolphin System.

### Goslon Yardstick Scores

Study models of the individuals with UCLP were retrieved from the archives at the centers. Each set of study models was given a unique number.

The researcher and another assessor were trained on the use of the Goslon Yardstick by the supervisor of the project. A replica of the 22 sets of study models of the master Goslon Yardstick was available during the training session and all the data collection sessions. These study models represent characteristics of occlusions for the five categories of the Goslon Yardstick.^21,25^ Guidelines to determine Goslon scores were discussed during the training session. The training session was conducted on a group of 20 sets of independently collected study models.

All three observers carried out the Goslon Yardstick scoring of the 49 study models in one sitting. Repeat scoring of 20 randomly selected study models was done two weeks later to determine intraobserver reliability and minimize recall bias. Inter- and intraobserver reliability was calculated using Cohen's Weighted Kappa statistic.^26^

### Statistical Analysis

The statistical analysis was descriptive and inferential. Demographic data, sidedness of the cleft, cephalometric data and the Goslon Yardstick scores for the individuals were entered into a Microsoft Excel (Raymond, WA) spreadsheet and transferred to SAS software (SAS Institute Inc, Cary, NC, USA Release 9.4) for statistical analysis. Correlations between cephalometric parameters (SNA, SNB, ANPog, SNB) and the Goslon scores were determined by linear regression analyses. The statistically significant level was set at *P* < .05.

The complete data set was used to assess interobserver reliability. Interobserver reliability was analyzed using Cohen's Weighted Kappa statistic. Landis and Koch^27^ recommend the following levels of agreement for categorical data: less than 0.2 (poor), 0.21–0.4 (fair), 0.41–0.60 (moderate), 0.61–0.80 (good), 0.81–1.00 (very good), and 1.00 (perfect agreement). Intraobserver reliability was assessed on a randomly selected subset of at least 20% of the total sample. Cephalometric data were obtained by randomly retracing 10 cephalograms by both observers and random rescoring of the Goslon Yardstick on 20 study models by the three observers two weeks after the previous session. This proportion was chosen based on established recommendations in the methodological literature, which suggest that reliability assessment on a representative subsample can provide sufficient evidence of measurement consistency while minimizing the burden of repeated measurements.^28^ This approach is commonly used in clinical and observational research where full-sample reliability testing is impractical or unnecessary. Intraobserver reliability of the cephalometric measurements was evaluated using the Concordance Correlation Coefficient (CCC).

## Results

### Patient Demographics

The age and gender characteristics of the sample are presented in Table 1. The number of males (16, 72.7%) and females (18, 66.7%) with left-sided clefts did not differ significantly (*P* = .760). There were 15 right-sided clefts (males 6, 27.3% and females 9, 33.3%), with no significant differences between the genders (*P* = .760).

**Table 1.** Age and Gender Distribution.

Age, years	Frequency (%)		
	Males	Females	All
7−8	1 (4.6)	6 (22.2)	7 (14.3)
9−10	11 (50.0)	8 (29.7)	19 (38.8)
11−12	7 (31.8)	6 (22.2)	13 (26.5)
13−14	3 (13.6)	7 (25.9)	10 (20.4)
Total	22 (100)	27 (100)	49 (100)
Mean (SD)	10.8 (1.79)	10.7 (2.20)	10.7 (2.01)
Median (IQR)	10 (10–12)	10 (9–13)	10 (10–12)
Min/Max	7/14	7/14	7/14

Abbreviations: SD, standard deviation; IQR, interquartile range; min, minimum; max, maximum.

### Cephalometric Parameters

The kappa statistics for interobserver reliability ranged from good (0.646) to very good (0.901). The CCC values for intraobserver reliability ranged from fair to good (for Li/ML = 0.5401 and “soft tissue” = 0.7880) to very good for the rest of the parameters (>0.85). In view of the agreement found between the measurements by the two observers, mean values of the two assessments were calculated for all the cephalometric parameters and used in all further analyses.

Descriptive statistics for the 14 cephalometric parameters are displayed in Table 2. There were no statistically significant differences between males and females for any of the cephalometric parameters (*P* > .05). When the cephalometric parameters were compared between left- and right-sided clefts, the only significant differences were for NSL/ML (*P* = .0108) and NSL/NL (*P* = .0234). There were no significant differences for any of the cephalometric parameters for left- and right-sided clefts in males, and only NSL/NL was significantly different between left- and right-sided clefts in females (*P* = .0396).

**Table 2.** Descriptive Statistics for Cephalometric Parameters for the Sample.

Parameter	Mean (SD)	Median (IQR)	Min /Max
SNA	77.5 (3.39)	77.9 (76.6–78.7)	65.6/88.9
SNB	77.3 (2.71)	77.6 (75.4–78.8)	69.7/83.9
SNPog	78.0 (2.60)	78.3 (76.2–79.6	72.0/84.2
ANB	0.2 (2.39)	1.0 (−1.2–2.0)	−6.1/5.0
ANPog	0.4 (3.50)	1.4 (−1.6–2.2)	−10.6/10.0
NSL/ML	34.9 (3.56)	35.6 (34.0–36.6)	18.7/42.1
NSL/NL	11.0 (1.94)	11.0 (9.6–11.8)	7.8/17.4
NL/ML	26.7 (3.93)	26.0 (24.4–29.7)	15.0/35.6
Is/FH	104 2 (7.43)	103.4 (98.2–109.7)	91.7/130.6
Is/NL	103.1 (6.74)	102.1 (98.6–105.6)	92.6/133.2
Ii/ML	90.2 (6.62)	90.8 (86.1–95.4)	75.1/105.1
Is/Ii	141.3 (11.42)	141.6 (133.7–150.4)	106.0/161.4
Nasolab	97.9 (9.57)	101.4 (90.8–105.6)	71.6/111.0
Soft tissue	171.2 (4.08)	171.5 (169.1–174.0)	161.1/178.7

Abbreviations: SD, standard deviation; IQR, interquartile range; Min, minimum; Max, maximum.

### Goslon Yardstick Scores

The Cohen's Weighted Kappa statistics for the interobserver reliability for the three observers ranged from good to very good (0.678, 0.750, 0.784). Intraobserver reliability ranged from good to very good (0.685, 0.699, 0.835).

The Goslon Yardstick scores for the three observers are presented in Table 3. The scores among the observers did not differ significantly (*P* = .554). The mean Goslon score was 2.89 (SD = 1.139). The mean score for males was 3.18 (SD = 1.201, *n* = 22) and for females was 2.66 (SD = 1.050, *n* = 27). The mean scores for males and females did not differ significantly (*P* = .113). There were also no statistically significant differences in Goslon scores between left- and right-sided clefts (*P* > .05).

**Table 3.** Goslon Yardstick Scores.

Score	Frequency (%)			
	Obs 1 *n* (%)	Obs 2 *n* (%)	Obs 3 *n* (%)	Combined *n* (%)
1	8 (16.3)	3 (6.1)	8 (16.3)	19 (12.9)
2	10 (20.4)	16 (32.7)	10 (20.4)	36 (24.5)
3	14 (28.6)	17 (34.7)	18 (36.8)	49 (33.3)
4	12 (24.5)	7 (14.3)	8 (16.3)	27 (18.4)
5	5 (10.2)	6 (12.2)	5 (10.2)	16 (10.9)
Total	49 (100)	49 (100)	49 (100)	147 (100)

Abbreviations: Obs, observer; *n*, number of individuals.

### Comparison of the Cephalometric Analysis and Goslon Yardstick

Linear regression analyses were performed for comparison of the (average) Goslon rating scores with the cephalometric parameters. The coefficients of determination, *R*^2^, were calculated. Percentagewise, the goodness-of-fit for SNA, SNB, ANB, and ANPog were 5.95%, 3.81%, 32.47%, and 21.69% respectively. The correlation coefficients for SNA, SNB, ANB, and ANPog were −0.2438 (*p* = .0913), 0.1952 (*p* = .1791), −0.5698 (*p* = .000), and −0.4657 (*p* = .0007), respectively.

The Goslon scores showed a statistically significant, moderate negative correlation with ANB (*r* = −0.5698) and a weaker negative correlation with ANPog (*r* = −0.4657).

## Discussion

The World Health Organization (WHO) promotes research to improve treatment outcomes of patients with CLP in randomized controlled trials (RCTs)^29^ in order to reduce the healthcare burden of these patients.^30,31^ There is evidence of a strong association between quality of treatment outcome and the availability of high-volume centralized care by dedicated teams.^4,14–17^ Researchers emphasise that CLP care in countries be centralized and standardized, and follow evidence-based, clinical practice recommendations to improve treatment outcomes for these individuals.^32–36^

In the United Kingdom, for example, the Clinical Standards Advisory Group (CSAG) advised that each comprehensive specialist team should be responsible for not less than 40 new cases for primary surgery annually.^37^ Many guidelines have been developed for high-income countries with adequate sources of finances, staffing and infrastructure and may, therefore, not be practical for implementation in SA.

Opinions differ regarding various surgical techniques and the timing of surgery in UCLP patients, and therefore, indicators of treatment outcome are useful for the treatment planning of individual patients and for the development of treatment protocols at CLP centers.^17,38–40^

### Cephalometric Analysis

Measurements of 14 angles on the cephalograms used in this study were similar to the cephalometric parameters used in the SC trials.^14^ There was different radiographic equipment at the two centers, and hence no linear measurements were analyzed. This is in line with other intercenter studies (Eurocleft^18^ and Americleft^16^) with measurements based on those used by Molsted et al. (1992).^17^

Possible problems using cephalograms as a means of assessing treatment outcomes in individuals with UCLP include abnormal anatomy,^25^ difficulty in identifying cephalometric landmarks,^25,41,42^ and lack of standardization of radiographic equipment in different centers.^40^ The point A can be difficult to assess in those with UCLP.^43,44^

Daskalogiannakis et al.^16^ and Urbanova et al.^15^ and Fudalej et al.,^45^ reporting on the Americleft and Slavcleft studies, respectively, have noted that it is important to take underlying skeletal patterns into consideration when intercenter studies are done, as the populations studied may exhibit differing craniofacial growth patterns.^15,16,45^ For example, class III skeletal patterns are more prevalent in other populations, including Malay^46^ and Japanese individuals.^47^ In our sample of UCLP, a class III skeletal pattern was observed.

Interobserver reliability between the two observers was good to excellent, depending on the parameters. The error of the method analysis (intraobserver reliability) for the skeletal sagittal measurements was fair to good, and although Li/ML = 0.5401) is relatively low, this did not influence our main outcome findings. These ranges compare favorably to those reported in other studies.^14,16^

The cephalometric data was pooled for males and females in this study because there were no statistically significant differences between males and females for any of the cephalometric parameters (*P* > .05). This is in line with other studies^12,48^ and those intercenter studies where cephalometric data were pooled.^14,16,18^

When right- and left-sided clefts were compared, there were statistically significant differences between the sides for NSL/ML and NSL/NL, with the mean values for the left-sided clefts being higher than for right-sided clefts. The prevalence of left-sided UCL/P is known to be about twice that of right-sided UCL/P in many populations.^49^ There were only 15 individuals with right-sided clefts in this study (9 females, 6 males), therefore, the results needed to be interpreted with caution. The relative influences of genetic and environmental factors on the development of UCLP, including sidedness of clefts, are topics that are currently receiving considerable attention.^50–54^

The large ranges for the cephalometric data evident in this study have also been reported in other studies of UCLP.^14^ Shaw et al.^8^ noted that considerable between-patients variation may exist within a population, despite the relative homogeneity of individuals with UCLP.

Table 4 shows the mean values of cephalometric data reported for this study, SC and some noncleft groups for purposes of comparison.^14,55^ This group and the SC group exhibited more maxillary retrusion (SNA) than the noncleft groups.

**Table 4.** Cephalometric Values for Males and Females From the WC Study, SC Study,^14^ and the Michigan,^55^ Nittedal^14^ and Rostock^14^ Growth Studies.

Cephalometric parameter	Gender	WC Mean age (10.7 years) 22 Male 27 Female Mean (SD)	SC Mean age (8.1 years) 262 Male 193 Female Mean (SD)	Michigan (10-year-olds) 46 Male 35 Female Mean (SD)	Nittedal (9-year-olds) 39 Male 35 Female Mean (SD)	Rostock (8-year-olds) 17 Male 16 Female Mean (SD)
SNA	Male	76.50 (3.73)	78.01 (3.98)	80.8 (3.1)	82.3 (3.0)	80.7 (3.7)
	Female	78.34 (2.89)	78.07 (4.23)	80.7 (3.7)	80.8 (3.4)	79.6 (3.2)
SNB	Male	76.42 (2.97)	75.0 (3.57)	76.5 (2.5)	78.9 (3.5)	77.0 (3.2)
	Female	78.02 (2.28)	75.53 (3.77)	76.7 (3.5)	78.0 (3.2)	75.7 (2.6)
SNPog	Male	77.25 (2.83)	75.61 (3.63)	76.9 (2.4)	n.a	76.7 (3.3)
	Female	78.59 (2.26)	76.28 (3.79)	77.2 (3.5)	n.a	75.5 (2.6)
ANB	Male	0.08 (2.74)	3.02 (3.39)	4.3 (2.0)	3.4 (2.1)	3.7 (2.0)
	Female	0.34 (2.10)	2.54 (3.37)	4.0 (2.7)	2.8 (2.2)	3.8 (1.8)
ANPog	Male	0.21 (4.01)	2.41 (3.62)	7.9 (4.9)	6.1 (4.9)	n.a
	Female	0.59 (3.10)	1.80 (3.77)	7.2 (5.7)	5.0 (5.4)	n.a
NSL/ML	Male	35.00 (4.63)	34.67 (5.29)	34.7 (4.7)	33.4 (5.9)	36.3 (4.6)
	Female	35.08 (2.23)	34.70 (5.42)	35.3 (5.1)	35.0 (4.6)	37.0 (3.2)
NSL/NL	Male	11.88 (3.98)	11.14 (4.00)	6.1 (2.6)	6.1 (2.8)	7.4 (3.1)
	Female	10.74 (2.02)	11.47 (3.92)	7.5 (2.8)	7.7 (2.5)	8.2 (3.7)
NL/ML	Male	27.68 (4.08)	23.57 (5.73)	28.5 (4.7)	n.a	27.5 (5.2)
	Female	25.37 (4.18)	23.19 (6.04)	27.8 (4.9)	n.a	27.2 (4.0)
Is/FH	Male	103.00 (6.73)	93.41 (8.29)	110.4 (6.7)	114.1 (4.7)	110.6 (4.9)
	Female	103.56 (12.29)	94.84 (8.07)	111.6 (7.1)	111.1 (5.0)	107.3 (5.0)
Is/NL	Male	101.50 (5.14)	94.91 (7.84)	111.1 (5.2)	n.a	n.a

Abbreviation: SD, standard deviation.

The mandible (SNB) was more prognathic in this group compared to the SC group. The individuals in this study presented with a Class III skeletal pattern, compared to the SC group and the noncleft groups.

It appears that the vertical growth pattern of the cleft groups does not differ much from that of the noncleft groups. The NSL/ML angle in this study group was statistically significantly larger in individuals with left-sided clefts compared to those with right-sided clefts, especially in females.

The mean values of NSL/NL are similar for the two cleft groups and much larger than for the noncleft groups. Kuseler et al.^14^ attribute the increased maxillary inclination to intrinsic factor/s related to growth associated with the cleft deformity, as increased maxillary inclination has been reported in unoperated individuals with UCLP^56^ and infants with UCLP before primary surgery has been done.^57^ In the study sample, the NSL/NL angle was statistically significantly larger in individuals with left-sided clefts compared to those with right-sided clefts. This finding needs further examination in future studies.

The lower incisor position (Is/ML) for the study group was more retroclined in males than in females. Both cleft groups had retroclined lower incisor positions relative to noncleft groups. The position of the lower incisor relative to the mandibular plane has been reported not to change significantly over time in individuals with UCLP who have not received orthodontic treatment.^58^

The mean values for the nasolabial angle are decreased in the cleft groups, more so in the study sample compared to the SC group. The decreased nasolabial angle may be caused by flattening of the tip of the nose^17^ and/or soft tissue characteristics of the population group in the WC. The soft tissue profile angles reflect the skeletal anatomy, with this angle being significantly smaller in both cleft groups compared to noncleft groups.

### Goslon Yardstick Scores

The Goslon Yardstick is a comprehensive validated rating system which includes many study model characteristics such as overjet, overbite and arch constriction.^59^ Sample sizes that are required for intercenter comparisons are realistic and achievable for many centers.^60^ Individuals with UCLP who have received orthodontic treatment and SABG should be excluded when assessing primary surgical outcomes, as their Goslon scores can be more favorable compared to individuals who have not received any treatment.^61,62^ The Goslon Yardstick has been reported to have good inter- and intraexaminer reliability.^19,22,25,47^

The mean Goslon score for the individuals with UCLP in this study was 2.89, with no statistically significant differences between the mean score for males and for females.

Several intercenter studies and other studies have used the Goslon Yardstick to measure DAR in individuals with UCLP of similar ages to this study. Goslon scores for some of the studies, including the six-center comparison study in England,^13^ the CSAG study,^63^ the Eurocleft study,^64^ the Slavcleft study,^45^ the Americleft study,^65^ and the SC trials^66^ are listed in Table 5. The Goslon results reported for the individuals with UCLP in this study compare favorably to some of those reported from other studies, for example, the SC,^66^ Americleft,^65^ Slavcleft^45^ studies and a study of Czech individuals.^61^ It is clear, however, that similar Goslon rating results are achieved by cleft centers globally using many different surgical treatment protocols. The various aspects related to primary surgery were not evaluated in this study.

**Table 5.** Summary of Goslon Scores From This Study and Some Previous Studies of Individuals With UCLP Aged 8–14 Years (No Orthodontic Treatment or SABG).

Study	Center	Mean Goslon score	SD	Age Years (SD)	n
Western Cape Van Zyl and Bellardie 2025 (Current study)	Two CLP centers, Western Cape, South Africa	2.89 1 = 12.9% 2 = 24.5% 3 = 33.3% 4 = 18.4% 5 = 10.9%		10.7 (2.01)	49
Galane and Bellardie 2024^67^	Western Cape, South Africa	2.91		11.5	68
Novakova et al. 2024^61^	Czech	2.89		10	28
Scandcleft Heliovaara et al. 2022^66^	9 North European cleft teams	2.90		8	411
Slavcleft Fudalej et al. 2019^45^	Warsaw Prague Bratislava	2.58 3.21 3.07	0.77 1.04 0.99	10.6 (1.3) 9.1 (0.9) 9.3 (1.8)	32 33 30
Southall,Waltersand Singer 2012^62^	Perth Australia	3.17	1.03	9	47
Americleft Hathaway et al. 2011^65^	A B C D E	3.38 3.66 2.63 3.33 3.18	0.86 0.61 0.81 0.88 0.66	9y4m 8y6m 8y10m 9y1m 9y2m	18 40 38 38 35
New Zealand Jack et al. 2011^68^	A B	3.5 3.1		9.9 (1.53) 9.6 (1.69)	28 31
Malaysia Zreaqat, Hassan and Halim 2009^45^		3.15 1 = 2.4% 2 = 24.4% 3 = 35.4% 4 = 31.1% 5 = 6.1%		8–10	82
Japan Susami et al. 2006^46^		3.5		8y4m	24
Warsaw (one-stage repair) Fudalej et al. 2009^69^	Warsaw compared to Oslo protocol	2.68 3.65	0.79 0.76	11.2 (1.65)	61
Sinko et al. 2008^22^	Vienna protocol	G1 + 2 = 71.5% G 3 = 19.6% G 4 + 5 = 8.9%		9.2	123
Eurocleft Molsted et al. 2005^64^	A B D E F A B D E F	2.6 2.5 3.5 2.6 3.0 2.1 2.1 3.1 2.5 3.2	0.6 0.7 0.9 0.8 0.8 0.6 0.7 1.1 0.7 0.9	9 9 9 9 9 12 12 12 12 12	23 26 25 30 19 23 26 25 30 20
CSAG Williams et al. 2001^63^	50 NHS centers, UK	3.14 Score 1 = 4% Score 2 = 30% Score 3 = 27% Score 4 = 26% Score 5 = 13%		12	229
Six-center international study Mars et al. 1992^13^	A B C D E F	2.59 3.46 3.04 3.03 2.64 2.47	0.76 0.92 0.87 0.75 0.64 0.66	10.13 (1.2)	24 27 24 25 30 19

Abbreviations: *n,* number of individuals in study; SD, standard deviation.

Shaw et al. (cited by Hathaway et al.^65^) reported that individuals with Goslon scores of 3.5 and higher would probably require orthognathic surgery to advance the maxilla at the end of their skeletal growth. Thirty-three per cent of individuals in the study group had a Goslon score of 3, and 29.26% had Goslon scores of 4 and 5. This means about 30% (Goslon 4,5) of the group will probably require orthognathic surgery at completion of growth, and a number of individuals with Goslon 3 scores may also require orthognathic surgery, depending on various factors, such as further maxillofacial growth and development, surgical procedures including SABG, and orthodontic treatment, which had not yet been done. Mars and Houston^56^ reported that the average Goslon score remains consistent through varying stages of dental growth and can be linked to the cephalometric analysis of these patients. Predictions of individuals who may require orthognathic surgery can be based on Goslon scores of 3.5, and higher.^65^ Buj-Acosta et al.,^70^ however, found a lack of evidence in the literature affirming the predictive validity of the Goslon Yardstick. The three studies they analyzed showed that the same Goslon categorization was maintained in 42.7%,^71^ 60%,^22^ and 64.7%^19^ of these long-term studies.

Studies reporting on DAR with sidedness of clefts are inconclusive.^49,53^ Fowler et al.^54^ advised that further studies with larger sample sizes of right-sided UCLP are required. They reported that DAR was clinically worse in right-sided UCLP, but the results could not be tested statistically because of the small sample size (left-sided UCLP = 80 and right-sided UCLP = 24). No significant differences were found for cleft sidedness and the Goslon scores for the group of individuals with UCLP in this group, but further studies with larger sample sizes should be attempted to investigate this further.

### Comparison of the Cephalometric Analysis and Goslon Yardstick Scores

In this study of individuals with UCLP presenting at the two specialist academic centers, a correlation coefficient of −0.5698 was found between ANB angle and the Goslon Yardstick scores, which was statistically significant (*P* = .000). Although the correlation coefficient is lower than that reported by Daskalogiannakis et al.^16^ for the Americleft study (*r* = −0.607), the trend is similar and supports the concept of using results of both the cephalometric analysis and Goslon scores to describe maxillary prominence. It was interesting to note that the correlation coefficient for the ANPog angle, relating point A to pogonion on the mandible, for the individuals with UCLP in the WC sample was −0.4657 (*P* = .0007).

### Limitations of This Study

The effect of dental anomalies on craniofacial morphology and DAR of individuals with UCLP was not taken into account in this study. It is known that the development of OFCs and dental agenesis has a genetic association.^72^ Moreover, if those with UCLP have two or more congenitally missing maxillary teeth, they will have a significantly smaller ANB angle compared to noncleft individuals.^73^ The prevalence data for UCLP missing incisors is known and indicates 10% of the sample might have been affected.^74^

The wide range of ages of the individuals in this study could contribute to the heterogeneity of growth characteristics observed. Differences in accessing CLP services, and therefore later presentation, are seen in deprived communities with limited access to care and transport.

This study did not include any information about the primary surgery techniques, timing of surgery, surgeons’ caseloads and skills and other associated factors related to the treatment of individuals born with UCLP.

## Conclusions

The results of this study provide evidence that the Goslon Yardstick is a valid and reliable indicator of sagittal jaw discrepancy.

The subjects UCLP in this study had a skeletal Class III relationship with no gender differences between the cephalometric parameters. The mean Goslon score for the sample was 2.89, which compares favorably to Goslon scores of DAR in some intercenter studies internationally. There were no statistically significant differences between males and females, or between right- and left-sided clefts for the Goslon scores.

These outcome measures are clinically relevant and will be used to constantly monitor services providing care for children born with CLP. They will be useful for intercenter studies within South Africa and in neighboring countries.
